# L-PGDS deficiency accelerated the development of naturally occurring age-related osteoarthritis

**DOI:** 10.18632/aging.202367

**Published:** 2020-12-23

**Authors:** Yassine Ouhaddi, Mehdi Najar, Frédéric Paré, Bertrand Lussier, Yoshihiro Urade, Mohamed Benderdour, Jean-Pierre Pelletier, Johanne Martel-Pelletier, Hassan Fahmi

**Affiliations:** 1 Osteoarthritis Research Unit, University of Montreal Hospital Research Center (CRCHUM), and Department of Medicine, University of Montreal, Montreal, QC H2X 0A9, Canada; 2 Faculty of Veterinary Medicine, Clinical Science, University of Montreal, Saint-Hyacinthe, QC, J2S 2M2, Canada; 3 Isotope Science Center, The University of Tokyo, Yayoi, Bunkyo-ku, Tokyo 113-0032, Japan; 4 Research Centre, Sacré-Coeur Hospital, University of Montreal, Montreal, QC H4J 1C5, Canada

**Keywords:** osteoarthritis, cartilage, aging, PGD2, L-PGDS

## Abstract

Osteoarthritis (OA) is the most common musculoskeletal disorder among the elderly. It is characterized by progressive cartilage degradation, synovial inflammation, subchondral bone remodeling and pain. Lipocalin prostaglandin D synthase (L-PGDS) is responsible for the biosynthesis of PGD_2_, which has been implicated in the regulation of inflammation and cartilage biology. This study aimed to evaluate the effect of L-PGDS deficiency on the development of naturally occurring age-related OA in mice.

OA-like structural changes were assessed by histology, immunohistochemistry, and micro–computed tomography. Pain related behaviours were assessed using the von Frey and the open-field assays.

L-PGDS deletion promoted cartilage degradation during aging, which was associated with enhanced expression of extracellular matrix degrading enzymes, matrix metalloprotease 13 (MMP-13) and a disintegrin and metalloproteinase with thrombospondin motifs 5 (ADAMTS-5), and their breakdown products, C1,2C, VDIPEN and NITEG. Moreover, L-PGDS deletion enhanced subchondral bone changes, but had no effect on its angiogenesis. Additionally, L-PGDS deletion increased mechanical sensitivity and reduced spontaneous locomotor activity. Finally, we showed that the expression of L-PGDS was elevated in aged mice. Together, these findings indicate an important role for L-PGDS in naturally occurring age-related OA. They also suggest that L-PGDS may constitute a new efficient therapeutic target in OA.

## INTRODUCTION

Osteoarthritis (OA) is the most common degenerative joint disorder, affecting more than one-third of adults aged 65 and older [1]. The major pathologic features of OA include progressive cartilage degradation, synovial inflammation, subchondral bone remodeling and pain [2, 3]. Although the exact molecular mechanisms underlying the pathogenesis of OA are not fully understood, a number of risk factors have been characterized, with aging and obesity being the most prominent [2, 3]. Other risk factors include joint injury, knee malalignment and genetics. Current treatments for OA aim to alleviate the symptoms such as pain and disability, but to date there are no proven treatment than can cure OA.

Progressive cartilage breakdown is a hallmark feature of OA and is predominantly mediated by proteolytic enzymes, most notably, a disintegrin and metalloproteinase with thrombospondin motifs 5 (ADAMTS-5) and matrix metalloproteinase 13 (MMP-13). Several studies reported that both enzymes play key roles in cartilage breakdown during OA. Deletion of the ADAMTS5 gene was shown to prevent cartilage cleavage in a joint instability model of OA [4]. Moreover, MMP-13 deficient mice are protected against [5], whereas MMP-13–transgenic mice develop, spontaneous OA-like cartilage damage [6].

Several studies suggest an important role for prostaglandin (PG) D2 in the pathophysiology of OA. PGD_2_ was shown to enhance chondrogenic differentiation, as assessed by increased expression of collagen type II and aggrecan [7, 8] and to prevent chondrocyte apoptosis [9]. Our group has demonstrated that treatment of human chondrocytes with PGD_2_ reduced IL-1-induced MMP-1 and MMP-13 expression, major effectors of cartilage breakdown during OA [10].

In addition to its anti-catabolic effects, PGD_2_ was reported to display anti-inflammatory properties. *In vitro* studies showed that treatment with PGD_2_ suppressed inflammatory responses in monocytes/macrophages [11], dendritic cells [12] and T cells [13]. PGD_2_ also has anti-inflammatory properties *in vivo*, and the administration of PGD_2_ was protective in several models of inflammatory conditions including chronic allergic lung inflammation [14], colitis [15], and atopic dermatitis [16]. The anti-inflammatory effect of PGD_2_ is further supported by the observation that overexpression of PGD_2_ synthase in mice attenuates, whereas its deletion exacerbates inflammation [17]. Finally, PGD_2_ was shown to suppress angiogenesis [18, 19], a key process in the pathogenesis of OA [20].

The biosynthesis of PGD_2_ from its precursor PGH_2_ is catalyzed by two PGD synthases (PGDSs): the lipocalin-type PGDS (L-PGDS; also called β-trace) and the hematopoietic PGDS (H-PGDS) [21]. L-PGDS is mainly expressed in the central nervous system [22], the heart [23], and the retina [24]. H-PGDS is essentially expressed in mast cells [25], megakaryocytes [26] and T cells [27].

We have shown that cartilage predominantly expresses L-PGDS [28, 29], however, the *in vivo* role of L-PGDS in the development of naturally occurring age-related OA is virtually unexplored. In the present study, we investigated the role of L-PGDS in the development of naturally occurring age-related OA using L-PGDS deficient mice.

## RESULTS

### L-PGDS deficiency accelerated cartilage degeneration with aging

First, we determined whether the lack of L-PGDS influences the expression of the major components of the extracellular matrix of articular cartilage, i.e. type II collagen and aggrecan. Real-time polymerase chain reaction (RT-PCR) analysis revealed that the levels of type II collagen and aggrecan mRNA in the knee joint of 3-month-old L-PGDS-/- mice were virtually similar to those of their wild type (WT) littermates (Supplementary Figure 1) indicating that L-PGDS deletion does not affect the expression of these two genes.

The body weight of L-PGDS-/- and WT mice was monitored throughout the experimental protocol. At 3 and 9 months, there were no differences in weight between L-PGDS-/- mice and their WT littermates. At 15 months, L-PGDS -/- mice were heavier than their WT littermates (Supplementary Figure 2A). EchoMRI analysis revealed that the differences in body weight at 15 months of age was due to greater fat mass in L-PGDS-/- mice (Supplementary Figure 2B).

**Figure 1:**
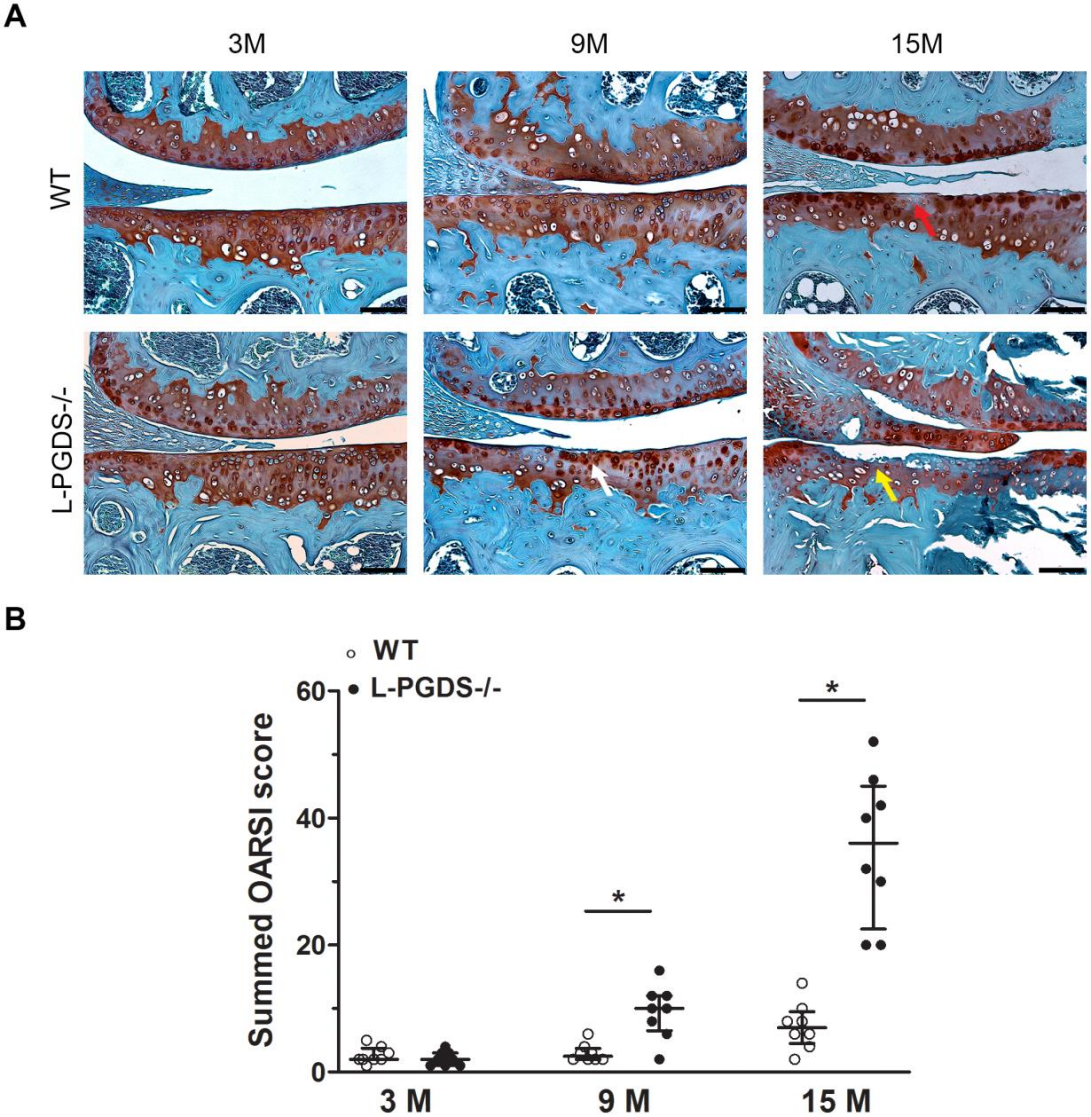
**Deletion of L-PGDS accelerated cartilage erosion with age.** (**A**) Coronal sections of whole knee joints from WT and L-PGDS-/- mice at ages 3, 9 and 15 months (n=8 mice/genotype/time point) were prepared and stained with Safranin O–fast green to assess the integrity of articular cartilage. The representative sections were selected based on the average score from each experimental group. Red arrow indicates loss of Safranin O staining. White arrow indicates areas of fibrillation and clefts. Yellow arrow indicates cartilage erosion. Scale bars=100 μM. (**B**) Summed histologic scores of knee cartilage from WT (open symbols) and LPGDS-/- (filled symbols) mice as determined using the OARSI scoring system. Results are presented as median with interquartile range. *P<0.05 versus WT mice.

To determine whether L-PGDS deletion alters OA development, we evaluated the integrity of articular cartilage in the knee joints of L-PGDS -/- mice and their WT littermates. At 3 months, there were no differences in the intensity of Safranin O staining or cartilage structure between L-PGDS-/- mice and their WT littermates (Figure 1A). At 9 months, moderate loss of safranin O staining was observed in the L-PGDS-/- articular cartilage, while such loss was not evidently observed in WT cartilage. Moreover, at this age, small fibrillation and clefts appeared in L-PGDS-/- cartilage, while there was no evidence of these changes in WT-type mice knees. At 15 months, L-PGDS -/- cartilage was severely fibrillated or eroded, while WT cartilage displayed only a moderate loss of safranin O staining and some small clefts (Figure 1A).

Quantification by the OARSI grading system [30] confirmed that OARSI scores were 4- and 5-fold higher (p < 0.05) in 9 and 15-month-old L-PGDS -/- mice, respectively, when compared with their WT littermates (Figure 1B). These data suggest that L-PGDS deficiency accelerated cartilage degeneration with aging.

### L-PGDS deficiency increased the expression of cartilage-degrading enzymes and their products

**Figure 2:**
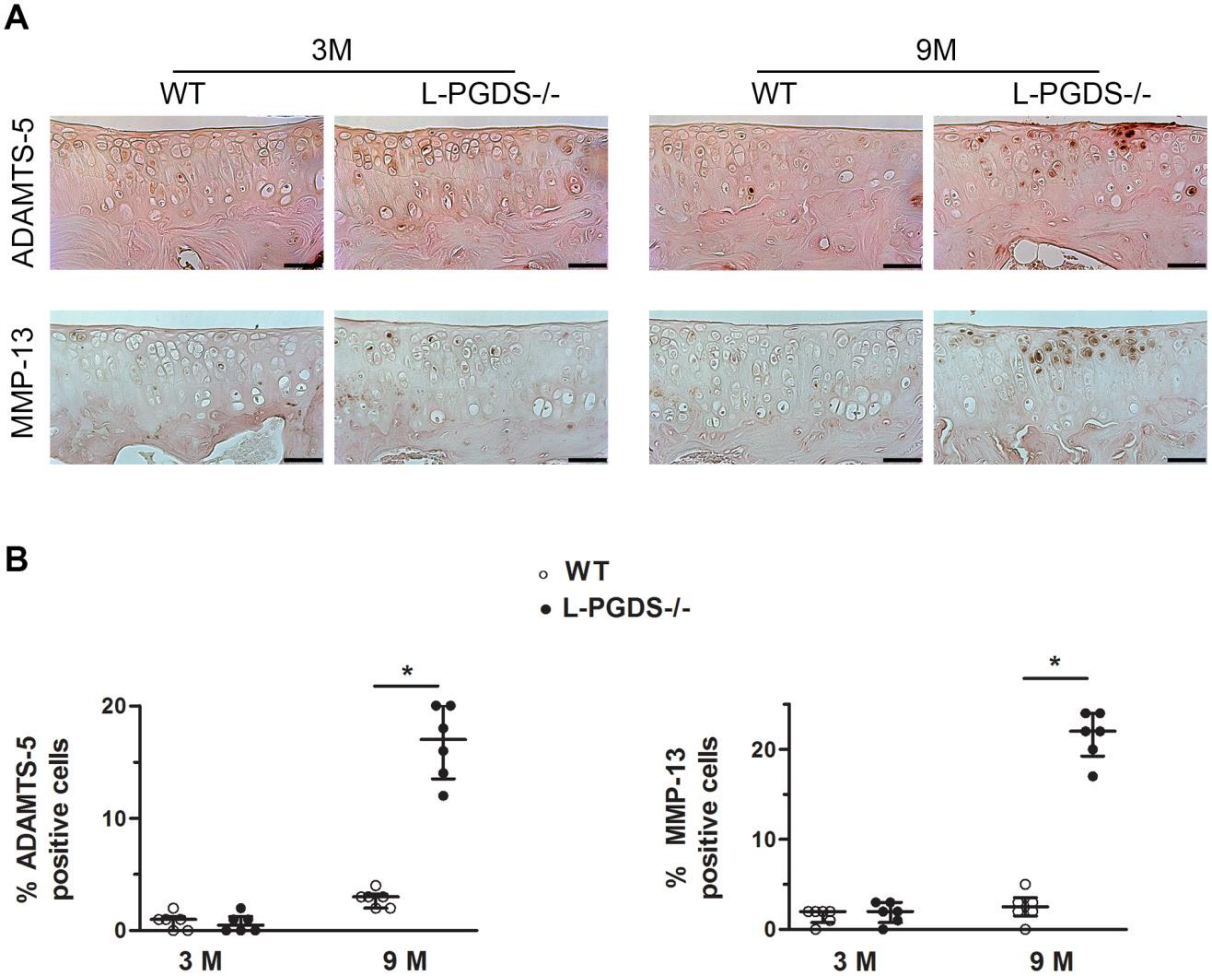
**L-PGDS deficiency enhanced MMP-13 and ADAMTS-5 expression in cartilage with age.** Knee joint sections from 3- (n=6 mice per genotype) and 9-month-old mice (n=6 mice per genotype) were analyzed by immunohistochemistry for MMP-13 and ADAMTS5 as described in the Materials and Methods section. (**A**) Representative images of immunohistochemical staining for ADAMTS5, and MMP-13 in knee joints from L-PGDS-/- and their WT littermates at 3 and 9 months of age. Scale bars=100 μm. (**B**) Percentage of chondrocytes expressing ADAMTS5, and MMP-13 in WT (open symbols) and L-PGDS-/- (filled symbols) mice. Data are presented as median with interquartile range of each group. *p<0.05 versus WT mice.

To define the mechanisms underlying cartilage degeneration in aged L-PGDS-/- mice, we analyzed the expression of key enzymes involved in cartilage degradation, i.e., MMP-13 and ADAMT-5 in the knee joints of L-PGDS-/-mice and their WT littermates at age 3 and 9 months. At 3 months, there was little or no staining for MMP-13 and ADAMTS-5 in the knee joints of L-PGDS-/- and WT mice (Figure 2). At 9 months, however, the number of cells staining for MMP-13 and ADAMTS-5 increased greatly (4- and 8-fold respectively, p < 0.05) in cartilage from L-PGDS-/- mice but remained very low in cartilage from WT mice (Figure 2).

We also analyzed the expression level of extracellular cartilage matrix degradation products: C1,2C, VDIPEN, and NITEGE. At 3 months, we observed only a weak staining for C1,2C, VDIPEN, and NITEGE in the articular cartilage of both genotypes (Supplementary Figure 3). At 9 months, the staining for either degradation product was greatly (4.5-, 5-, and 1.5-fold respectively, p < 0.05) enhanced in cartilage from L-PGDS-/- mice and very weak in cartilage from WT mice (Supplementary Figure 3). These data suggest the loss of L-PGDS accelerates cartilage degradation likely via increased expression of MMP-13 and ADAMTS-5.

### L-PGDS deletion promoted synovitis in aged mice

**Figure 3:**
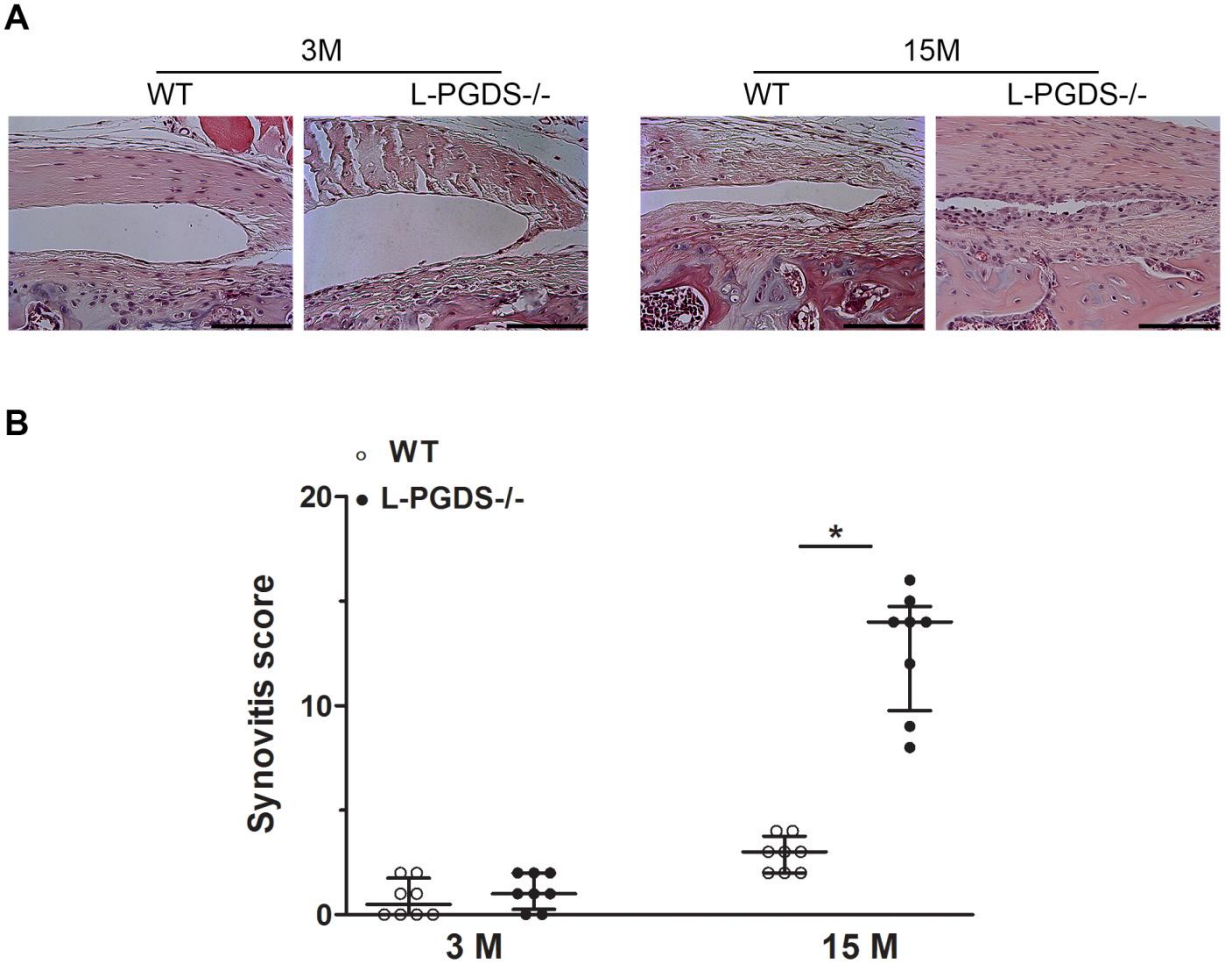
**Deletion of L-PGDS increased synovitis in aged L-PGDS-/- mice.** Coronal sections of whole knee joints from WT and L-PGDS-/- mice at ages 3, 9 and 15 months (n=8 mice/genotype/time point) were prepared and stained with hematoxylin and eosin to asses synovium changes. (**A**) Representative hematoxylin and eosin staining, and (**B**) summed synovitis scores of WT (open symbols) and L-PGDS-/- (filled symbols) mice at ages 3, 9, and 15 months. Scale bars=100 μm. The representative sections were selected based on the average score from each experimental group. Results are presented as median with interquartile range. *p<0.05 versus WT mice.

We also analyzed the effect of L-PGDS deletion on synovial changes during aging. At 3 months, there was no difference between L-PGDS-/- mice and their WT littermates for hyperplasia or synovial cell density (Figure 3A). At 15 months, the synovium of WT mice showed only minor changes. In contrast, the synovium of L-PGDS-/- mice showed marked thickening and hyperplasia (Figure 3A). Semiquantitative scoring confirmed a significant increase (4.5-fold, p < 0.05) in severity of synovitis in L-PGDS-/- mice at 15 months of age compared with WT mice (Figure 3B).

### L-PGDS deficiency increased OA-like bony changes in aged L-PGDS KO mice

**Figure 4:**
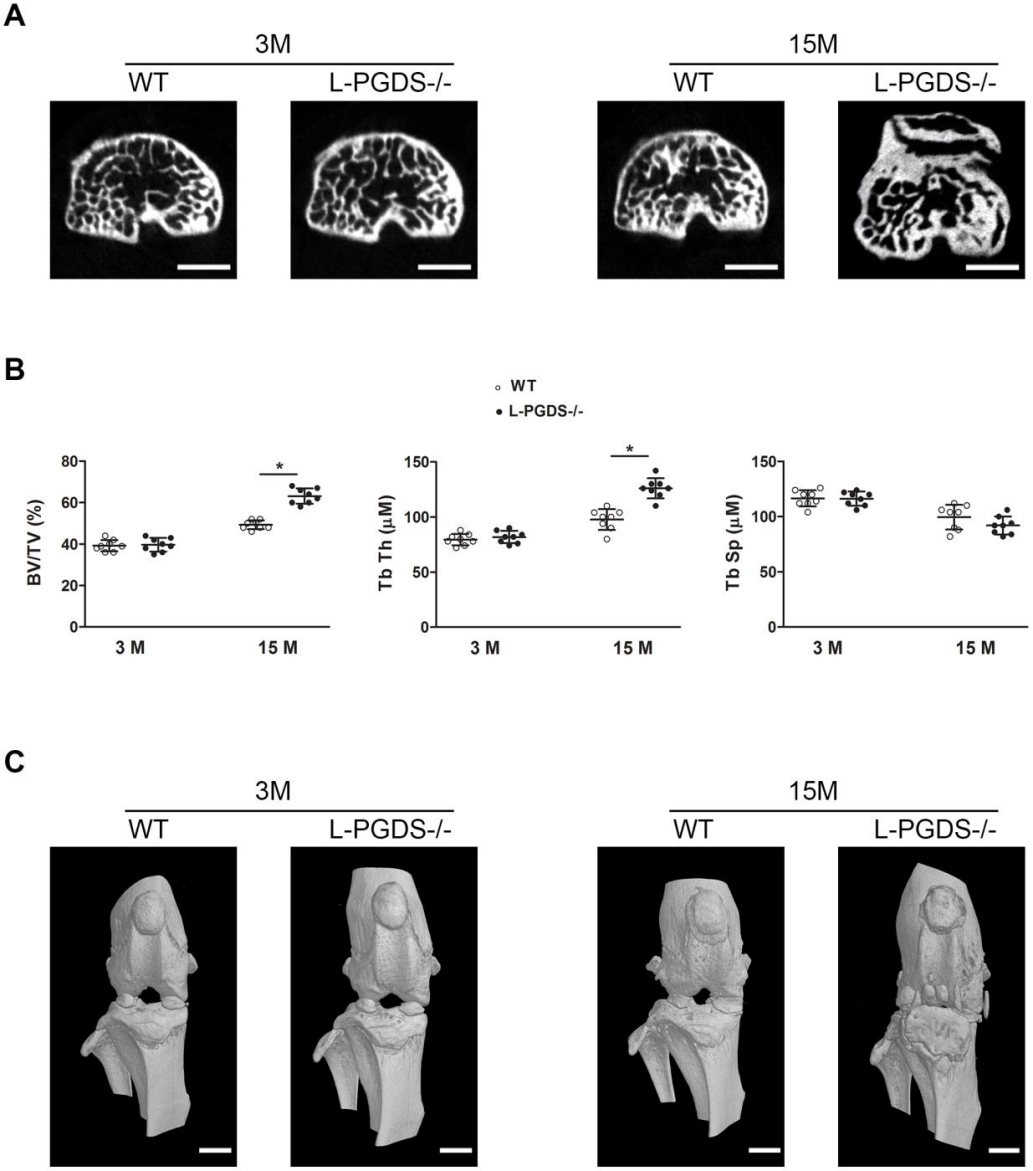
**Micro-CT analysis of the subchondral bone of the tibial plateau of WT and L-PGDS-/- mice at 3 and 15 months.** Knee joints from 3- and 15-month-old WT and L-PGDS-/- mice (n=8 mice/genotype/time point) were evaluated by micro-CT. (**A**) Representative axial micro-CT images of the subchondral bone compartment. Scale bars=1 mm. (**B**) Quantification of BV/TV, Tb.Th, and Tb.Sp in the subchondral bone region of the medial tibial plateau of WT (open symbols) and L-PGDS-/- (filled symbols) mice. Data are presented as mean ± SD. *p<0.05 versus WT mice. (**C**) Representative 3D reconstructions of the knee joints of WT and L-PGDS-/- mice at ages 3 months and 15 months PGDS-/- mice. Scale bars=1 mm.

Subchondral bone changes play a crucial role in the pathogenesis of OA [2, 3]. We, therefore, used micro-CT to analyse subchondral bone changes at 3 and 15 months in WT and L-PGDS-/- mice. As illustrated in Figure 4A, subchondral bone sclerosis was found at the medial tibia of both WT and L-PGDS-/- mice at 15 months, relative to 3 months, although L-PGDS-/- mice sclerosis seemed higher.

Quantification of subchondral bone microarchitectural parameters at 3 months showed that there was no difference in bone volume over total volume (BV/TV), trabecular thickness (Tb.Th) and trabecular separation (Tb.Sp) between WT and L-PGDS-/- mice (Figure 4B). At 15 months, however, BV/TV and Tb.Th of L-PGDS-/- mice were significantly higher (28 and 30 %, respectively, p < 0.05) those of WT mice. In contrast, Tb.Sp was slightly (8%) lower (Figure 4B).

Three dimensional reconstructions of the knee in L-PGDS-/- mice at 15 months showed OA-like bony changes, including osteophyte formation, joint space narrowing, meniscal calcification, and periarticular ectopic bone formation. In contrast, their WT counterparts at the same age showed unimpaired morphological features (Figure 4C).

### L-PGDS deficiency does not affect subchondral bone angiogenesis

**Figure 5:**
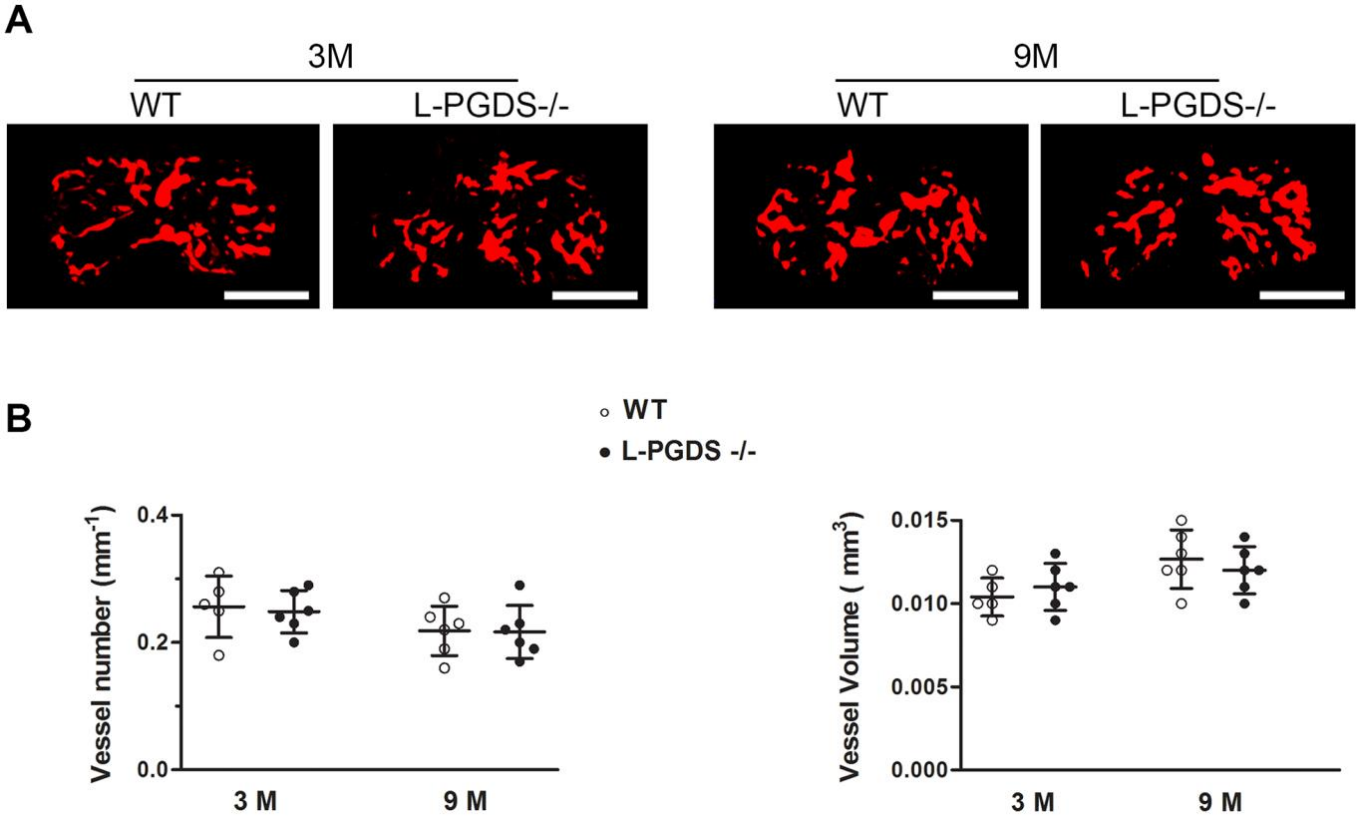
**Micro-CT-based microangiography of the tibial subchondral bone of WT and L-PGDS-/- mice at 3 and 9 months.** Subchondral bone angiogenesis in WT and L-PGDS-/- mice at 3 (n = 5 for WT, n = 6 for L-PGDS-/-) and 9 months (n = 6 per genotype) of age was evaluated by micro-CT angiography. (**A**) Representative 3D micro-CT-based micro-angiography of tibial subchondral bone at 3 and 15 months. Scale bars=1 mm. (**B**) Quantification of vessel volume (VV) and vessel number (VN) in the subchondral bone region of WT (open symbols) and L-PGDS-/- (filled symbols) mice. Data are presented as mean ± SD.

Previous studies reported that PGD_2_ has anti-angiogenic properties [18, 19] and abnormal angiogenesis is a known pathological feature of OA. We therefore used Microfil contrast-enhanced micro-CT-based microangiography to assess angiogenesis in subchondral bone of WT and L-PGDS-/- mice. As shown in Figure 5, the number and volume of blood vessels in subchondral bone were not different between L-PGDS-/- and WT mice at 3 months of age. These parameters were also not different between both genotypes at 15 months.

Angiogenesis at the osteochondral junction is also an important factor in the pathogenesis of knee OA. Using hematoxylin and eosin staining we found no evidence of osteochondral angiogenesis at the medial tibial plateaux of L-PGDS-/- mice and their WT littermates at 3 and 15 months of age (data not shown). These results suggest that L-PGDS deletion does not affect subchondral bone angiogenesis and likely does not aggravate OA via enhanced angiogenesis.

### L-PGDS deficiency enhances pain-related behaviours

**Figure 6:**
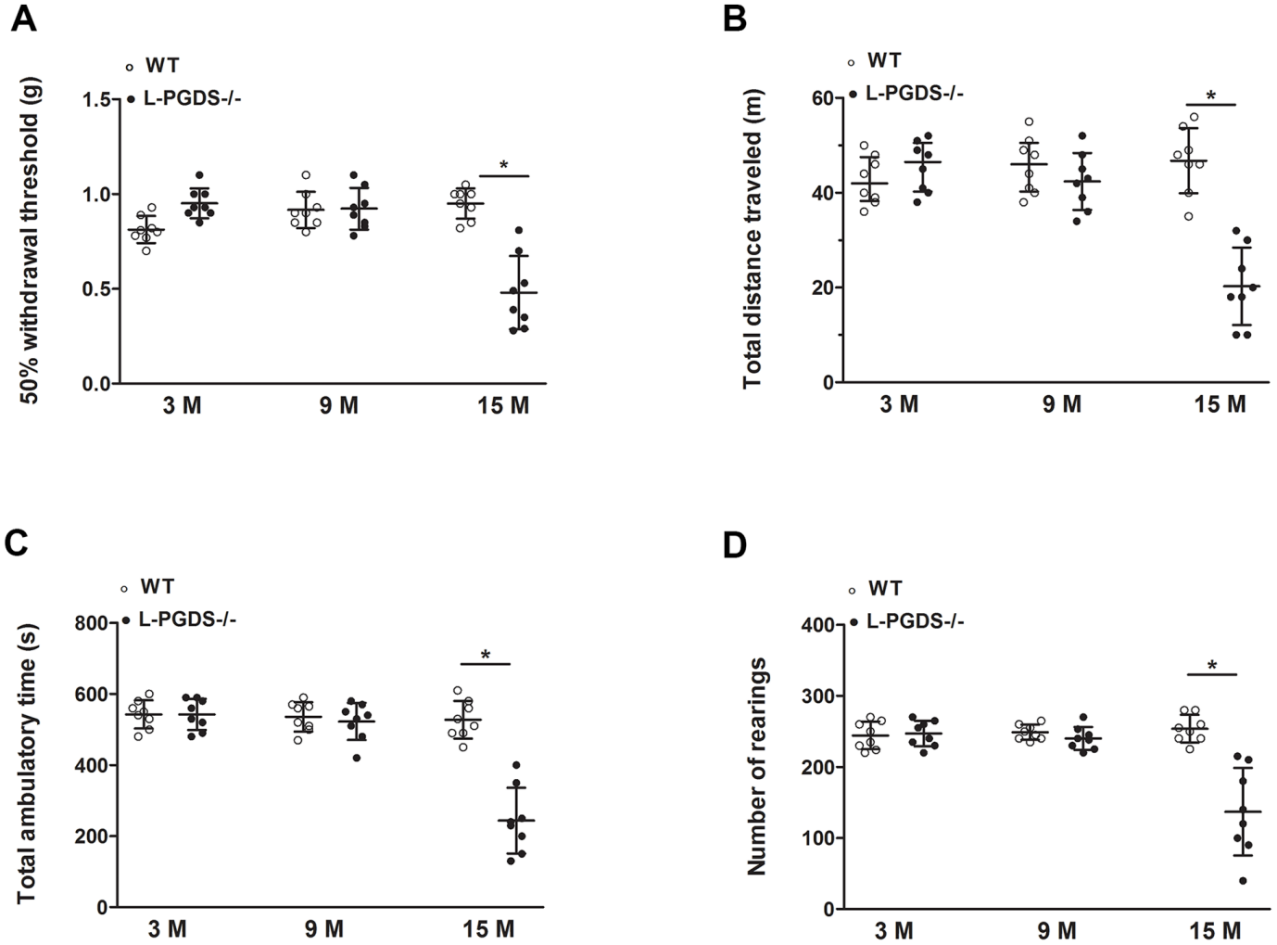
**Pain-related behaviours during aging in WT and L-PGDS-/- mice.** (**A**) Mechanical allodynia (50% paw withdrawal threshold (PWT)) in WT and L-PGDS-/- mice at 3, 9 and 15 months (n = 8 mice/genotype/time point) of age. Total distance travelled (**B**), ambulatory time (**C**) and number of rears (**D**) during 1 h testing period. Data are presented as mean ± SD. *p<0.05 versus WT mice.

To further investigate the relationship between L-PGDS deletion and OA, we compared OA-related pain (mechanical allodynia) in L-PGDS-/- and WT mice using the von Frey filament assay. At 3 months, L-PGDS-/- mice showed a slightly reduced (~20%) mechanical sensitivity, as indicated by higher paw withdrawal thresholds, compared to their WT littermates (Figure 6A). At 9 months, there was no significant difference between L-PGDS-/- and WT in mechanical sensitivity. At 15 months, however, L-PGDS-/- mice showed higher mechanical sensitivity (~50%), compared to age-matched WT controls (Figure 6A). Thus, L-PGDS deletion causes increased mechanical sensitivity in aged mice.

We also examined whether L-PGDS deletion impairs locomotor activity (non-evoked pain-like behaviour). We evaluated various standard parameters including total distance travelled, ambulatory time and rearing (standing on hind limbs). At 3 and 9 months, spontaneous locomotor activity parameters were similar in L-PGDS -/- and their WT littermates (Figure 6B–6D). In contrast, at 15 months L-PGDS -/- mice displayed reduced total distance travelled, ambulatory time and rearing (60, 54 and 46%, respectively, p < 0.05) compared to WT mice (Figure 6B–6D). These data suggest that L-PGDS deletion reduced spontaneous locomotor activity by 15 months.

### Up-regulation of L-PGDS during ageing in WT mice

**Figure 7:**
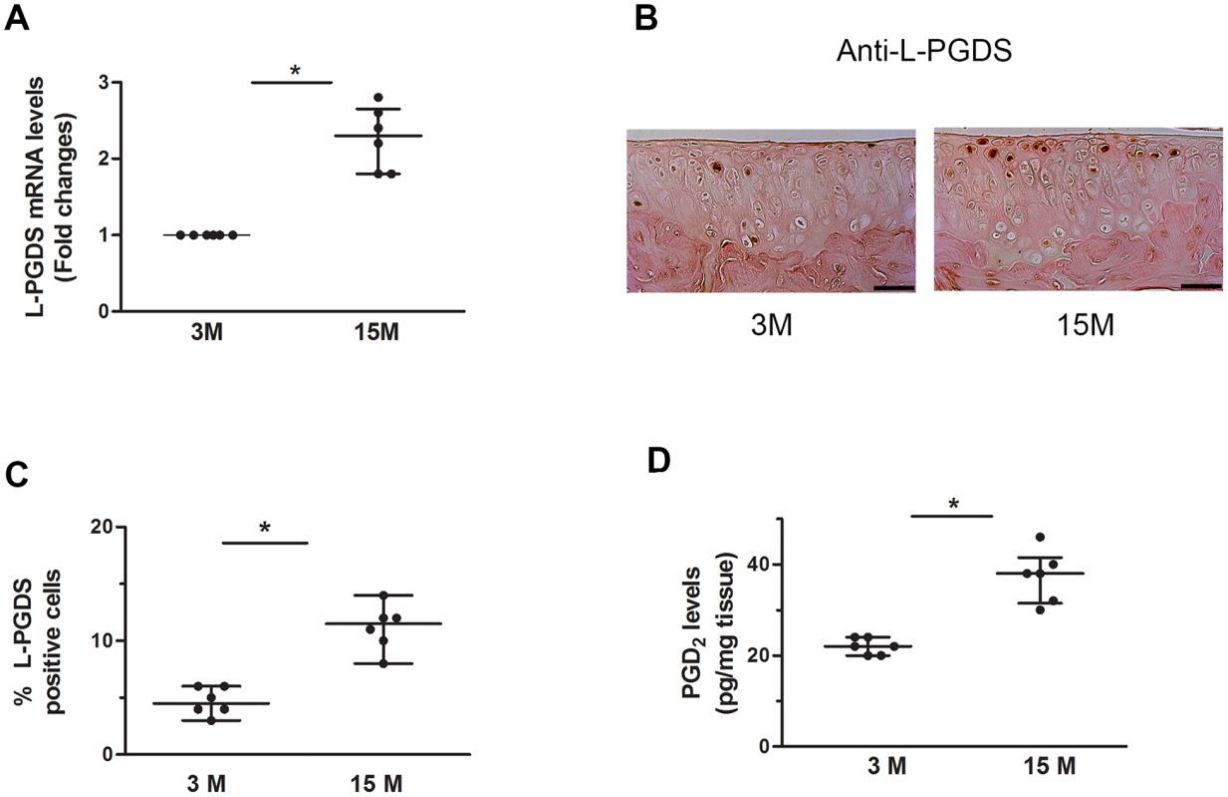
**Increased expression of L-PGDS in cartilage of aged WT mice.** (**A**) Total RNA was extracted from the joints of 3- and 15-month-old mice (n=6 mice/genotype/time point), and the levels of L-PGDS mRNA were determined by real-time RT-PCR. Results are expressed as -fold change, considering the value for 3-month-old mice as 1*.* (**B**) Representative images of immunohistochemical staining for L-PGDS in knee joints from of 3- and 15-month-old mice. Scale bars=100 μm. (**C**) Percentage of chondrocytes expressing L-PGDS in cartilage (n=6 mice/genotype/time point). Results are shown as median with interquartile range. (**D**) PGD_2_ levels in knee joint of 3- and 15-month-old mice (n=6 mice/genotype/time point), as determined by ELISA. *p<0.05 versus 3-month-old mice.

Finally, we examined the expression level of L-PGDS mRNA in the knee joint of WT mice at 3 and 15 months of age. Results are expressed as -fold change, considering the value of control animals (3-month-old mice) as 1. We found that the level of L-PGDS mRNA was significantly increased (2.2-fold, p < 0.05) in the joint of 15-month-old mice compared to those of 3-month-old mice (Figure 7A).

To determine whether the observed changes in the mRNA levels were paralleled by changes in the protein levels, we performed immunohistochemical analysis. As shown in Figure 7B, 7C, the levels of L-PGDS protein was also increased (2.4-fold, p < 0.05) at 15 months of age. Moreover, the level of PGD2 was increased (1.8-fold, p < 0.05) in the knee of 15-month-old mice (Figure 7D). Hence, the expression level of L-PGDS in cartilage increased in aged WT mice.

## DISCUSSION

In the present study, we showed for the first time that L-PGDS plays an important role in the development of naturally occurring age-related OA. We demonstrated that L-PGDS deletion promotes cartilage degradation during aging, and that it was associated with enhanced expression of cartilage degrading enzymes, MMP-13 and ADAMTS-5, and their breakdown products, VDIPEN, C12C and NITEG. In addition, L-PGDS deletion enhanced subchondral bone changes and mechanical sensitivity, and reduced locomotor activity. Finally, we showed that the expression of L-PGDS was elevated in aged WT mice. These findings suggest that L-PGDS has protective properties in OA and may constitute an attractive therapeutic target.

To define the role of L-PGDS in aging associated-OA, we analyzed age-related structural changes in the knee joints of L-PGDS-/- and their WT littermates. We found that L-PGDS-/- mice displayed enhanced and accelerated cartilage degeneration with age and was associated with increased expression of MMP-13, and ADAMTS-5 and their degradation products, VDIPEN, NITEG and C12C. This is in accordance with our recent findings showing that L-PGDS deletion enhanced the expression of MMP-13 and ADAMTS-5 and cartilage degradation in a mouse model of instability-induced OA [31]. Similarly, deletion of DP1, a PGD_2_ receptor, leads to increased expression of MMP-13 and ADAMTs and exacerbated cartilage damage in both aging associated and instability-induced OA [32]. The increased level of cartilage degrading enzymes in L-PGDS-/- mice is also in agreement with our previous *in vitro* studies showing that PGD_2_ prevented the expression of MMP-13 and MMP-1 in cultured chondrocytes and cartilage explants [10].

In the present study, aged L-PGDS-/- mice exhibited increased body weight, suggesting that excess mechanical stress may contribute to the disease in aged L-PGDS -/- mice. It is well known that overweight is associated with the incidence and progression of OA, and weight loss was found to reduce the risk for OA, relieve symptoms, and improve functions in human OA [33, 34]. Similarly, animal studies showed that overweight increased the severity of aging-associated OA [35] and instability-induced OA [36, 37]. Increased weight enhances the mechanical load of the weight-bearing joints, leading to alterations in the composition, structure, metabolism, and mechanical properties of articular cartilage, subchondral bone, and other joint tissues, and consequently promoting the degeneration of articular cartilage [38].

In addition to increasing body weight, adipose tissue is the source of a wide variety of pro-inflammatory cytokines termed adipokines. One adipokine, leptin, is known to play a key role in the onset and progression of OA. For example, treatment with leptin, alone or in synergy with IL-1, induced cartilage degradation via upregulation MMP-1 and MMP-13 expression [39]. Treatment with leptin was also reported to induce the production of several pro-inflammatory mediators known for their role in the pathogenesis of OA, including NO, IL-1, IL-6 and IL-8 [40]. Injection of leptin into the knee joint of rats increased the expression of several cartilage degrading enzymes including MMP-1, MMP-3, MMP-13, ADAMTS-4 and DAMTS-5 [41]. Griffin et al showed in leptin-deficient (ob/ob) and leptin receptor–deficient (db/db) mice that adiposity is insufficient to induce OA in the absence of leptin signaling [42]. Moreover, serum and synovial leptin levels are increased in OA patients [43, 44]. It should be noted that the circulating levels of leptin were reported to increase in aged L-PGDS-/- mice [45]. Thus, elevated levels of leptin could contribute to the development of OA in aged L-PGDS-/- mice.

We also observed a marked synovitis in aged L-PGDS-/- mice. This is probably due to the anti-inflammatory effects of L-PGDS and is in agreement with previous finding showing that L-PGDS metabolites, PGD_2_ [46] or 15-PGJ2 [47], attenuate synovitis in inflammatory arthritis. Moreover, treatment with PGD_2_ or with its analog BW245, was reported to reduce inflammatory responses in experimental models of allergic lung inflammation [14], colitis [15], and atopic dermatitis [16]. It is also possible that the observed synovitis in aged L-PGDS-/- mice is exacerbated by increased body weight. Previous studies have shown that obesity aggravates synovitis in aging-associated [48] and surgically-induced [49] OA. Finally, the observed synovitis in aged L-PGDS-/- mice may also be due to increased leptin levels. Some studies reported that intraarticular injection of leptin increased synovial hyperplasia in collagen-induced arthritis in mice [50], and enhanced leptin levels were shown to be associated with synovitis in human OA [51]. Thus, in the present study, it seems that loss of L-PGDS results in not only cartilage degradation but also in increased synovial inflammation1synovitis.

We also investigated the effect of L-PGDS deficiency on subchondral bone changes during aging. Micro-CT analysis revealed that aged L-PGDS-/- mice had enhanced subchondral bone sclerosis, osteophyte formation, periarticular ectopic bone formation, increased mineralization of the meniscus and joint space narrowing. Bony changes in L-PGDS-/- mice might likely have resulted from lower levels of PGD_2_. Several studies showed that PGD_2_ plays important roles in bone metabolism. PGD_2_ inhibits osteoclastogenesis and bone resorption [52], promotes osteoclast apoptosis [53], stimulates osteoblast calcification [54], and prevents ovariectomy-induced decreased bone mineral density [55]. The increased mechanical stress due to obesity could also contribute to subchondral sclerosis and bony changes in aged L-PGDS-/- mice. Another factor that may be involved in bone remodeling in L-PGDS-/- mice is the increased levels of leptin, which have both direct and indirect effects on bone metabolism [56].

Pain is the most common symptom of OA and a primary reason for patients with OA to seek medical attention. In the present study, we showed that young L-PGDS-/- mice displayed reduced responses to mechanical stimuli, which concurs with a previous study showing that L-PGDS deletion attenuated PGE_2_-induced allodynia [57]. In contrast, aged L-PGDS-/- mice displayed mechanical hypersensitivity (enhanced responses to mechanical stimuli). These findings suggest that the effect of L-PGDS deletion on mechanical sensitivity is age-dependent, attenuating pain in young mice, but enhancing it in aged mice. A context dependent effect of PGD_2_ on pain has been previously reported by Telleria-Diaz et al, who demonstrated that topical application of PGD_2_ to the spinal cord of normal knee rats had no effect on responses to mechanical stimulation of the knee joint. In contrast, these responses were decreased in inflamed knee joints [58].

The observed pain behaviour in aged L-PGDS-/- mice may also be caused by increased body weight and is consistent with previous studies showing a functional relationship between obesity and OA pain. Obesity and overweight are strongly associated with onset and exacerbation of pain [59], and weight loss decreases the pain as well as the risk of OA [34]. Leptin can also participate in pain behaviour in aged L-PGDS-/- mice. Indeed, leptin was reported to critically contribute to neuropathic allodynia in rats [60]. Moreover, leptin-deficient mice showed reduced tactile allodynia, which was reversed by the administration of leptin [61].

In addition, we showed that aged L-PGDS -/- mice exhibited decreased locomotor activity compared to WT mice, suggesting that reduced physical activity could also contribute to OA exacerbation in L-PGDS **-/-** mice. Indeed, reduced physical activity has been reported to aggravate OA in obese mice [35].

Angiogenesis contributes to the initiation and progression of OA, and PGD_2_ was reported to have anti-angiogenic properties [18, 19]. In the present study, we found that the number and volume of blood vessels in subchondral bone were not different between L-PGDS-/- and WT mice at 3 and 15 months of age. Additionally, vascular invasion into calcified cartilage at the osteochondral junction was not obvious in aged L-PGDS-/- mice and their WT littermates. Although we cannot exclude that there may be differences in angiogenesis that we did not detect with the assays used in the present study, these results suggest that the acceleration of OA development in L-PGDS deficient mice is not due to enhanced subchondral bone angiogenesis.

Our inability to detect vascular changes in aged OA L-PGDS-/- mice contrasts with previous data showing subchondral neovascularization in mouse models of OA [62, 63] and osteochondral vascularization in aged mice [64]. The reasons for these discrepancies are unclear but are most likely due to differences in experimental design. Indeed, the studies reporting increased subchondral neovascularization were performed with surgical models [62, 63], which are more severe than the aging model used in the present study. The study reporting angiogenesis at the osteochondral junction was performed with 20-24 month-old mice [64], while in the present study we utilized 15-month old mice.

Finally, we showed that the expression of L-PGDS was up-regulated in aged mice, suggesting that although protective, the increased level of L-PGDS is not enough to optimally attenuate the development of OA. This is in agreement with previous findings reporting that L-PGDS [65, 66] levels increased with age. This is also consistent with our findings showing increased L-PGDS expression in human [28] guinea pig, dog [29] and mouse [31] OA cartilage. Increased expression of L-PGDS was also reported in other age-related conditions, such as glaucoma [67] and atherosclerosis [68]. The observed elevated levels of L-PGDS in cartilage could be induced by mechanical loading and/or inflammatory factors. Both mechanical loading and pro-inflammatory cytokines were reported to up-regulate L-PGDS expression in chondrocytes [28, 69].

This study has some limitations. First, we used only male mice because age-related OA is more prevalent and more severe in males than females [70, 71]. Second, we did not evaluate food intake and energy expenditure. Further studies are warranted to define the exact role of weight gain, fat content and leptin in the pathogenesis of OA in aged L-PGDS -/- mice. Third, while our data suggests that L-PGDS deletion accelerates the development of OA via up-regulation of the key cartilage degrading enzymes ADAMT-5 and MMP-13, the molecular mechanisms underlying these processes are not fully unraveled. More research will be required to shed light on the exact molecular and cellular mechanisms underlying the acceleration and exacerbation of OA in L-PGDS-/- mice. Finally, although our findings clearly demonstrate that L-PGDS deletion accelerated OA development, it is not clear whether this was due to L-PGDS loss in cartilage, bone or both, because L-PGDS is expressed in both chondrocytes and osteoblasts. In addition, L-PGDS is present in many tissues, suggesting that loss of L-PGDS in other tissues could also contribute to the exacerbation of OA. Further studies using mice with tissue specific deletion of L-PGDS may be needed to fully understand the mechanisms by which L-PGDS deficiency promotes OA.

These findings indicate an important role for L-PGDS in naturally occurring age-related OA. They also raise the possibility that the induction of L-PGDS pathway could be an attractive new strategy for the treatment of OA, as well as various other arthritic diseases.

## MATERIALS AND METHODS

### Mice

All animal experiments were approved by the Institutional Animal Protection Committee of the University of Montreal Hospital Research Centre (CRCHUM), and performed in accordance with the Animal Research Reporting of *in Vivo* Experiments (ARRIVE) guidelines [72]. L-PGDS-/- mice were generated as described previously [57]. In these mice, the L-PGDS gene was disrupted by replacing a 1.84-kb fragment containing parts of exons II-V with the neomycin resistance gene. L-PGDS-/- mice were backcrossed onto the C57BL/6 background for 10 generations. L-PGDS-/- and WT mice used in these experiments were generated by breeding heterozygous littermates, and genotypes were identified by PCR of tail biopsy DNA extract.

Mice were maintained under standard pathogen-free conditions and a 12-hour light/dark cycle, with water and a pelleted standard normal diet (catalog no. 2918; Teklad Global, Harlan Laboratories, Indianapolis, IN, USA) made available ad libitum. Mice were housed individually in filter-top cages (38 x 20 x 15 cm in dimension). Cotton nestlets and hard plastic tubes were placed in each cage for environmental enrichment. The mice appeared healthy and showed normal behaviour throughout the study. Nine mice were excluded from this study due incomplete decalcification (n=3) or inadequate perfusion (n=6).

After behavioural tests, mice were sacrificed at 3, 9 and 15 months of age. Knees were harvested, and subjected to micro-CT, histological and immunohistochemical analyses.

### Body composition

The body fat content of the mice was determined by magnetic resonance imaging using EchoMRI (Echo Medical Systems, Houston Scientific, Houston, TX, USA).

### Histological evaluation of osteoarthritic changes

The harvested knee joints were fixed in TissuFix (Chaptec, Montreal, QC, Canada), decalcified in 10% EDTA for 14 days at 4° C, and embedded in paraffin. Coronal sections (5 μm) were obtained through the entire joint at 80 μm intervals and stained with Safranin O–fast green (eight sections per joint) or hematoxylin and eosin (five sections per joint). Cartilage damage was assessed in accordance with the recommendations of the Osteoarthritis Research Society International (OARSI) guidelines [30]. Synovitis was assessed using a synovitis scoring system which evaluate the enlargement of the synovial lining cell layer on a scale of 0-3 (0 = 1-2 cells, 1 = 2-4 cells, 2 = 4-9 cells and 3 = 10 or more cells) and cellular density in the synovial stroma on a scale of 0-3 (0 = normal cellularity, 1 = slightly increased cellularity, 2 = moderately increased cellularity and 3 = greatly increased cellularity) [73]. All sections were graded by two scorers (YO and MN) in a blinded manner. The four quadrants (medial tibial plateau, medial femoral condyle, lateral tibial plateau, and lateral femoral condyle) of the knee were assessed, and the scores were added to obtain the summed histologic score.

### Immunohistochemistry

Knee joints were fixed in TissuFix, decalcified in 10% EDTA for 14 days at 4° C, and embedded in paraffin. Immunohistochemical analysis was performed as previously described [31, 32, 74]. Briefly, sections (4 sections per joint) from the weight bearing area were pre-incubated with chondroitinase ABC (0.25 U/ml in PBS pH 8.0) for 60 min at 37° C, followed by a 30 min incubation with Triton X-100 (0.3%) at room temperature. Slides were then washed in phosphate-buffered saline (PBS) followed by 2% hydrogen peroxide/PBS for 15 min. They were further incubated for 45 min with 2% normal serum (Vector Laboratories, Burlingame, CA) and overlaid with the primary antibody for 18 hours at 4° C in a humidified chamber. The following antibodies were used: rabbit polyclonal anti-L-PGDS (1:200 dilution; Cayman Chemical, Ann Arbor, MI), rabbit polyclonal anti-ADAMTS5 (1:100 dilution; Cedarlane, Hornby, ON), rabbit polyclonal anti-MMP-13 (1:100 dilution; Sigma-Aldrich), rabbit polyclonal anti-C1,2C (1:500 dilution; IBEX Technologies, Mont-Royal, QC, Canada), rabbit polyclonal anti-VDIPEN (1:800 Gladys dilution; a generous gift from Dr. J. Mort, Hospital for Children, McGill University Hospital Centre, Montreal, Quebec, Canada), and rabbit polyclonal anti-NITEG (1:100 dilution; Novus Biologicals, Littleton, CO). Each slide was washed 3 times in PBS (pH 7.4) and incubated with a secondary antibody using the Vectastain ABC kit (Vector Laboratories) following the manufacturer’s instructions. The color was developed with 3,3’-diaminobenzidine (DAB) (Vector Laboratories) containing hydrogen peroxide. The slides were counterstained with eosin. The specificity of the staining was confirmed by substituting the primary antibody with a non-specific IgG from the same host as the primary antibody.

For ADAMTS-5, MMP-13 and NITEG staining, the total number of chondrocytes and the number of chondrocytes staining positive were evaluated and results were expressed as the percentage of chondrocytes staining positive (cell score). For C1,2C and VDIPEN staining, images were captured at 250X with a Leitz Diaplan microscope connected to BIOQUANT OSTEO 2012 software. Surface area of positively stained extracellular cartilage matrix was measured, and data expressed as % of positive stained area over total area. Each slide was examined and scored by 2 independent observers (MN and YO), who were blinded to group allocation.

### Micro-CT analysis of bone

The knee joints were scanned using the SkyScan 1176 micro-CT scanner (SkyScan, Aartselaar, Belgium) at 50 kV, 500 μA, with a pixel size of 9 μm and a 0.5-mm aluminum filter. Data were recorded at every 3-degree rotation step through 180 degrees. Image slices were reconstructed using NRecon software (version 1.6.3.2, SkyScan). The region of interest (ROI) included the area between the epiphyseal growth plate and the articular cartilage. The following morphometric parameters: BV/TV, Tb.Th. and Tb.Sp. were determined using CT-Analyser software (SkyScan). CTVox software (SkyScan) was used to create 3-D images.

### Micro-CT-based microangiography

Blood vessels in subchondral bone were imaged by angiography of Microfil-perfused bones as previously described [63]. Mice were anesthetized with isoflurane, the thoracic cavity was opened, and inferior vena cava was severed. The vascular system was flushed with 0.9% normal saline containing heparin sodium (100 U/ml) at a flow rate of 0.5 ml/minute via a needle inserted into the left ventricle.

The specimens were then pressure fixed with 10% neutral buffered formalin, which was washed off (from the vessels) with heparinized saline solution. The vasculature was then injected with a radiopaque silicone rubber compound containing lead chromate (Microfil MV-122; Flow Tech Inc., Carver, MA, USA), and the bodies were stored at 4° C overnight to allow full polymerisation. The hind limbs were isolated and fixed in 10% neutral buffered formalin for five days and decalcified in RDO Rapid Decalcifier (Apex Engineering Products Corporation, Aurora, IL, USA) for two hours. The specimens were scanned using SkyScan 1176 micro-CT scanner with the resolution of 9 μm isotropic voxel size. The region of interest (ROI) began below the subchondral plate and extended for 0.3 mm distally. Histomorphometric parameters including vessel volume (Vess. Vol), and vessel number (Vess. Nb) were evaluated using CT-Analyser software, while 3D images were created using CTVox software.

### Mechanical allodynia

Mechanical allodynia was measured according to the method described by Chaplan et al [75]. Mice were acclimatized to a metal mesh grid for two hours prior to testing, and a calibrated set of von Frey filaments (Stoelting Co., Wood Dale, IL, USA) was applied to the plantar surface of the hind paw and was maintained for up to six seconds. The 0.16 g filament was always the first stimulus. A rapid withdrawal of the hind paw was recorded as a positive response. The force of the von Frey filament was increased or decreased following a negative or positive response, respectively. The 50% paw withdrawal threshold was determined twice on each hind paw and averaged, with sequential measurements made at five-minute intervals.

### Locomotor activity

Mice were acclimated to the testing room for two hours before open-field testing. Locomotor activity was assessed using the VersaMax Animal Activity Monitoring System (AccuScan Instruments, Columbus, OH, USA). Mice were placed into the center of individual chambers (29 x 22 x 22 cm) and allowed free exploration for 60 minutes. The following parameters were measured: total distance travelled, ambulatory time, and rearing.

### Statistical analysis

Sample size calculations were based on our primary outcome "OA histopathology" and our previous studies [32, 74]. Our sample size would provide > 80% power to detect a 50% change in mean OARSI scores with a significance level of p=0.05. Histological and immunohistochemical data were assessed using the Mann-Whitney U test (for comparison of two groups), or Kruskal-Wallis followed by Dunn’s multiple comparisons test (for comparison between more than two groups). Subchondral bone, blood vessels and behavioural (mechanical allodynia and locomotor activity) data were analyzed using Student’s t-test (for comparison of two groups) or one-way ANOVA followed by Bonferroni's multiple comparisons test (for comparison between more than two groups). *P*-value < 0.05 was considered significant. All analyses were performed using Prism 8.0 (GraphPad Software, San Diego, CA).

## Supplementary Material

Supplementary Figures
