# Supplementary material for: L-PGDS deficiency accelerated the development of naturally occurring age-related osteoarthritis
Source: Aging (Albany NY). 2020 Dec 23;12(24):24778–97. doi: 10.18632/aging.202367 (PMC7803483; doi:10.18632/aging.202367)
Supplement: Supplementary Figures [file aging-12-202367-s001.pdf]

SUPPLEMENTARY FIGURES

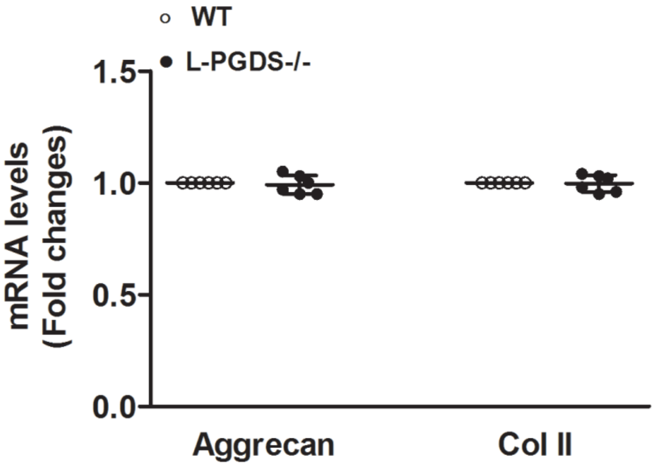

Supplementary Figure 1. Expression of aggrecan and Col II mRNA in WT and L-PGDS-/- joints. Total RNA was extracted from the joints of three-month-old L-PGDS-/- mice and their WT littermates (n=6 per genotype), and the levels of aggrecan and Col II mRNA were determined by real-time RT-PCR. Results are expressed as -fold change, considering the value for WT mice as 1. *p<0.05 versus WT mice.

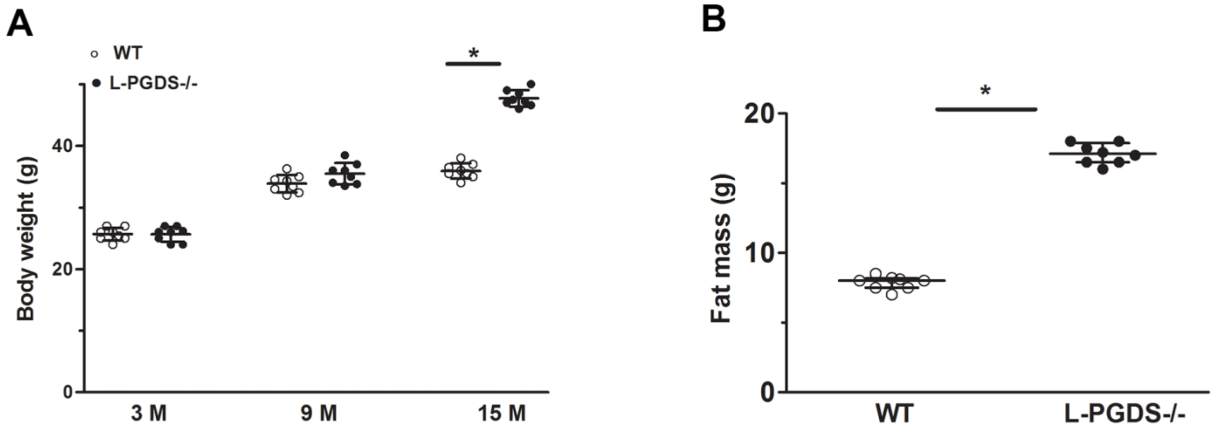

Supplementary Figure 2. Body weight and composition/fat content/fat mass of WT and L-PGDS-/- mice. (**A**) Body weight of WT and L-PGDS-/- mice at 3, 9 and 15 months of age (n=8 mice/genotype/time point). (**B**) Fat mass/ Percentage of body fat of WT and L-PGDS-/- mice at 15 months of age (n=8 mice/genotype). Data are presented as mean±SD. *p<0.05 versus WT mice.

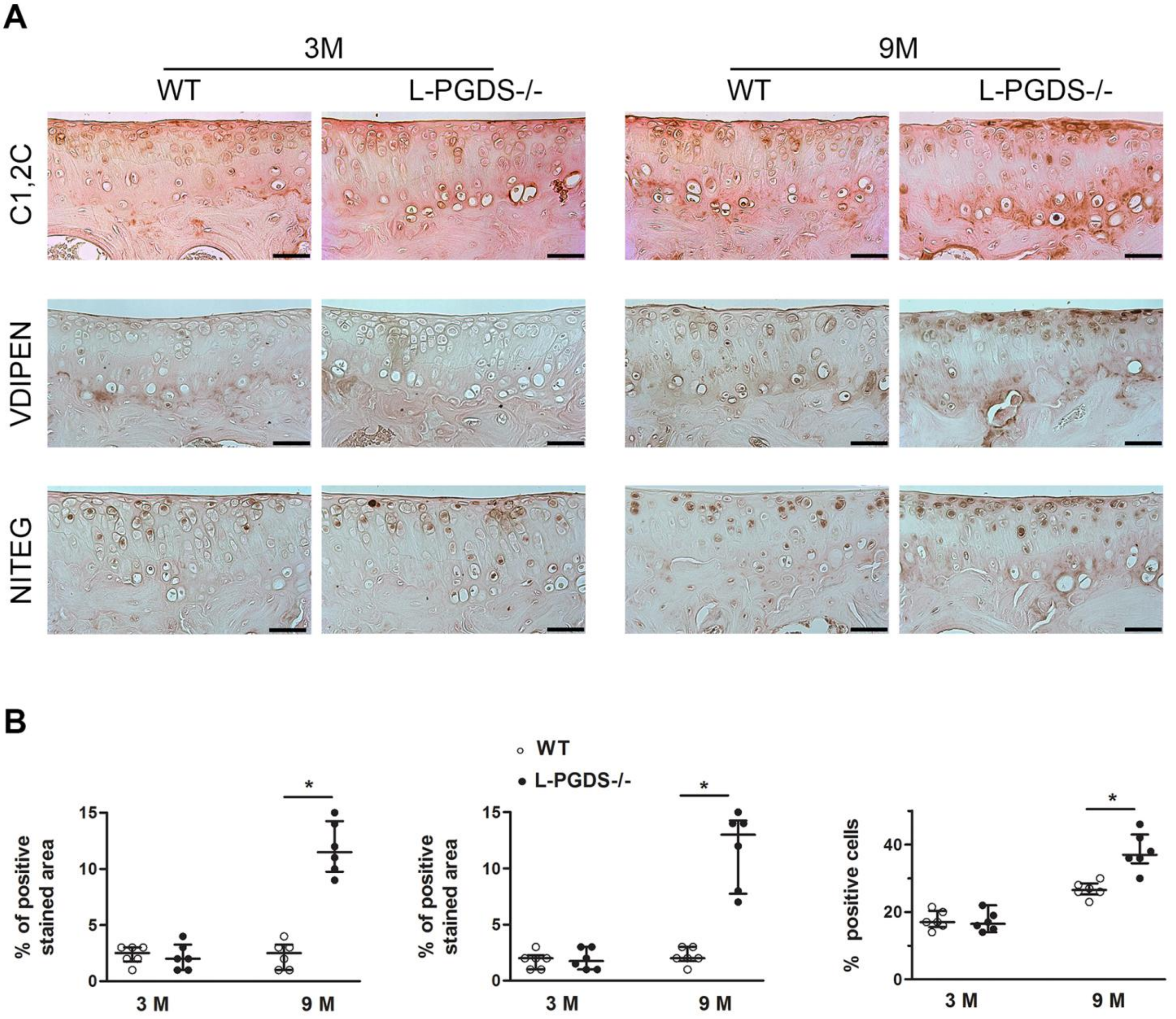

Supplementary Figure 3. L-PGDS deletion enhanced the expression of C1, 2C, NITEG and VDIPEN in cartilage. Knee joint sections from 3- (n=6 mice per genotype) and 9-month-old mice (n=6 mice per genotype) were analyzed by immunohistochemistry for C1,2C, NITEG and VDIPEN, as described in the Materials and Methods section. (**A**) Representative images of immunohistochemical staining for C1,2C, NITEG and VDIPEN. Scale bars=100 µm. (**B**) Percentage of positive stained area (C1,2C and VDIPEN), and positive chondrocytes (NITEG) in WT (open symbols) and L-PGDS-/- (filled symbols) mice. Data are presented as median with interquartile range. *$p<0.05$ versus WT mice.
